# MiRNAs and miRNA Polymorphisms Modify Drug Response

**DOI:** 10.3390/ijerph13111096

**Published:** 2016-11-08

**Authors:** Mu-Peng Li, Yao-Dong Hu, Xiao-Lei Hu, Yan-Jiao Zhang, Yong-Long Yang, Chun Jiang, Jie Tang, Xiao-Ping Chen

**Affiliations:** 1 Department of Clinical Pharmacology, Xiangya Hospital, Central South University, Changsha 410008, China; elskesunny@163.com (M.-P.L.); huxiaolei66@126.com (X.-L.H.); zhangyj287112687@163.com (Y.-J.Z.); JChun213@163.com (C.J.); 2 Institute of Clinical Pharmacology, Hunan Key Laboratory of Pharmacogenetics, Central South University, Changsha 410078, China; 3 Department of Cardiology, Heping Hospital Affiliated to Changzhi Medical College, Changzhi 046000, China; leexz2010@163.com; 4 Haikou People’s Hospital and Affiliated Haikou Hospital of Xiangya Medical School, Central South University, Haikou 570311, China; yyl0756@yeah.net

**Keywords:** microRNA, drug response, miRNA polymorphisms, prognosis

## Abstract

Differences in expression of drug response-related genes contribute to inter-individual variation in drugs’ biological effects. MicroRNAs (miRNAs) are small noncoding RNAs emerging as new players in epigenetic regulation of gene expression at post-transcriptional level. MiRNAs regulate the expression of genes involved in drug metabolism, drug transportation, drug targets and downstream signal molecules directly or indirectly. MiRNA polymorphisms, the genetic variations affecting miRNA expression and/or miRNA-mRNA interaction, provide a new insight into the understanding of inter-individual difference in drug response. Here, we provide an overview of the recent progress in miRNAs mediated regulation of biotransformation enzymes, drug transporters, and nuclear receptors. We also describe the implications of miRNA polymorphisms in cancer chemotherapy response.

## 1. Introduction

Drug responses are complex traits determined by both genetic and environmental factors. Variations in expression of drug response-related proteins such as drug-metabolizing enzymes (DMEs), drug transporters and therapeutic mechanisms (including drug targets and downstream signal molecules) are the important source of inter-individual variability in drug response. In spite of genetic factors, epigenetic modification of gene expression at transcriptional and post-transcriptional levels contributes to variations in the expression of drug response related genes [1]. Accumulated evidence has shown that genes encoding DMEs, drug transporters, nuclear receptors, and drug targets are under epigenetic control [1,2]. Pharmacoepigenomics, a newly emerged field of combined study on genetic variations and epigenetic modifications in drug response, is supposed to expand the scope of pharmacogenomics and may provide more definite answers to the role of environmental factors in variable drug response [3].

In addition to transcriptional regulation of gene expression by DNA methylation, histone modification and xenosensor modulation, microRNAs (miRNAs) mediated post-transcriptional regulation is a newly recognized mechanism of gene expression regulation that attracts much interest in recent years [4]. Understanding of the miRNA-related mechanisms in drug response opens a new field in pharmacogenetics and pharmacoepigenomics. In this review, we focused on miRNA and its prospects in pharmacogenomics and translational medicine. PubMed, EMBASE, and Web of Science databases were searched up to July 2016 for studies that evaluated associations between miRNAs as well as miRNA polymorphisms and drug response. Relevant publications were identified by searching for combinations of “miRNAs”, “drug metabolizing enzymes”, “drug transporters”, “miRNA polymorphisms”, “drug response” and their synonyms.

## 2. MiRNAs: A New Player in Gene Function and Drug Response

**Figure 1:**
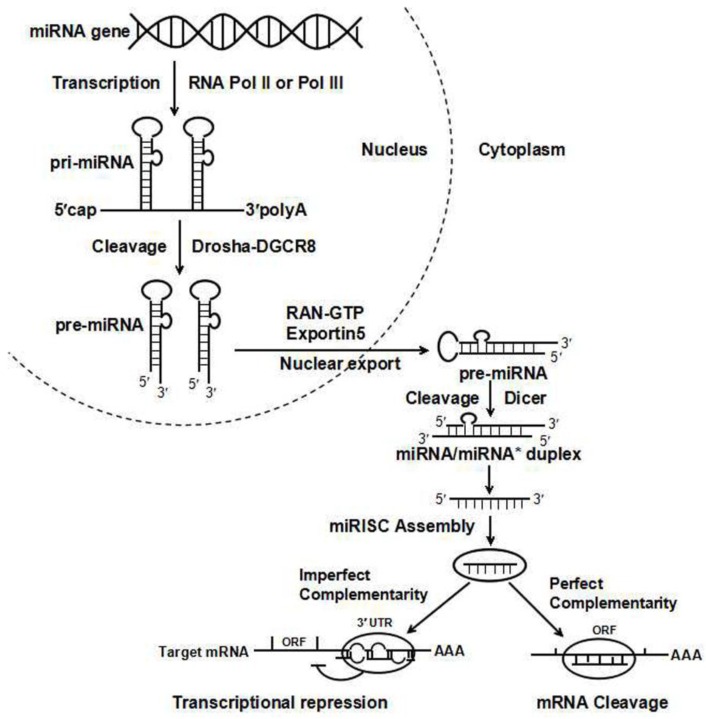
MiRNA biogenesis and posttranslational silencing mechanism. The miRNA maturation includes the production of the primary miRNA transcript (pri-miRNA) by RNA polymerase II or III and cleavage of the pri-miRNA by Drosha in the nucleus. Pre-miRNAs are transported to the cytoplasm by Exportin 5 and are processed into miRNA/miRNA* duplexes by Dicer. Only one strand of the miRNA/miRNA* duplex is processed into the RNA-induced silencing complex (RISC), which subsequently acts on its target mRNAs through mRNA cleavage, translational repression or deadenylation, depending on the level of complementarity between the miRNA and its targets. ORF, open reading frame.

MiRNAs bind to complementary regions of the target transcripts and regulate gene expression via translational repression or mRNA degradation (Figure 1). Up to date, about 1881 miRNAs are identified in human genome [5]. However, only 523 human miRBase entries are robustly supported as miRNA genes [5]. Given miRNAs function as important regulators of a wide range of cellular processes, identification of canonical miRNA becomes particularly important. It is estimated that more than 60% of human protein-coding genes harbor miRNA target sites in their 3’-untranslated regions (3’-UTRs), and miRNAs are predicted to control about 30% of human genes [6]. Roles of miRNAs in organ development and pathogenesis of human diseases have been extensively studied in recent years [7]. The expression of some DMEs, drug transporters, and drug targets can also be regulated by miRNAs through direct or indirect mechanisms [8]. MiRNA-mediated gene regulation provides new insight into the understanding of variations in an individual’s response to therapeutic drugs.

## 3. MiRNAs-Mediated Regulation of DMEs and Drug Transporters

Numerous studies have demonstrated that miRNAs can regulate DMEs and drug transporters, including cytochrome P450s (CYP450s), ABC and SLC transporters, and xenobiotic receptors (Table 1).

### 3.1. Direct Repression of DME by miRNAs

CYP450s superfamily, the largest group of phase I enzymes, catalyze a huge diversity of drugs in adult human liver. MiRNA-mediated epigenetic regulation is observed to affect CYP450 expression [8].

CYP1A1 is responsible for the metabolisms of carcinogenic metabolites, such as benzo(a)pyrene. A positive correlation between miR-18b as well as miR-20b and CYP1A1 mRNA levels was observed in immortalized lymphoblastiod cell lines [9]. However, miR-18b and miR-20b exhibited no correlation with CYP1A1 mRNA and protein levels in human liver tissue [10]. Moreover, luciferase assays revealed that CYP1A1 is a direct target of miR-892a [11]. A significant negative correlation was observed between miR‑892a level and CYP1A1 protein expression [11]. Recently, mRNA and protein levels of CYP1A1 exhibited negative correlation with miR-132, miR-142-3p, and miR-21 in a cohort of 92 human liver tissue [10].

CYP2A13 is critical for the metabolic activation of the tobacco-specific carcinogen 4-(methyl-nitrosamino)-1-(3-pyridyl)-1-butanone (NNK), a potent and tobacco-specific procarcinogen [12]. CYP2A3 is the orthologue of human CYP2A13 in rats. In a study mimicking the early stages of lung cancer development in rats under chronic NNK exposure, Kalscheuer et al., observed that the expression of several miRNAs, such as miR-101, miR-126*, miR-199 and miR-34, was decreased, while the expression of CYP2A3 was increased in the early stage of tumorigenesis, and reporter assays showed *CYP2A3* is a direct target of miR-126* [13].

CYP1B1 is involved in metabolism of several procarcinogens and chemotherapeutic drugs such as doxorubicin. It was the first reported CYP450 isoenzyme undergoing miRNA regulation. The expression levels of miR-27b [14] and miR-200c [15] correlated reversely with CYP1B1 protein in breast cancerous and renal cell cancer tissues, respectively. As miR-27b exhibits lower levels in breast cancer, the miR-27b mediated translational inhibition of CYP1B1 expression may account for the tumor-specific expression of CYP1B1 at protein level rather than mRNA level in breast cancer [16]. Upregulation of CYP1B1 due to miR-27b and miR-200c downregulation may thus lead to decreased drug response in cancer.

CYP2C8 is involved in the detoxification of more than 60 clinical drugs. Transfection of miR-103 or miR-107 precursors decreases CYP2C8 protein level via MRE within the *CYP2C8* 3’-UTR in primary human hepatocytes [17]. Furthermore, inhibition of these miRNAs results in an increase in CYP2C8 protein expression [17]. In addition, miR-21, miR-27a, miR-142-3p, miR-223, and miR-539 exhibited reverse correlation with CYP2C8 mRNA level in human liver tissue [10].

CYP2C9 is responsible for metabolism of about 20% of clinically used drugs, such as warfarin and phenytoin. In a human liver tissue, CYP2C9 mRNA level exhibited negative correlation with numerous miRNAs, of which the most significant miRNAs were miR-16, miR-17, miR-29a, and miR-28-3p [10]. Furthermore, overexpression of miR-128-3p could suppress CYP2C9 mRNA and protein expression in HepaRG cells [18]. MiR-128-3p expression was inversely correlated with *CYP2C9* mRNA expression in hepatocellular carcinoma (HCC) tumor tissues [18]. And luciferase reporter assay revealed that *CYP2C9* was targeted directly by miR-128-3p [18].

CYP2C19 is a monooxygenase which metabolizes many clinically prescribed therapeutic agents, including selective serotonin reuptake inhibitors, proton pump inhibitors, clopidogrel, citalopram, diazepam, and imipramine. Overexpression of miR-103 and miR-107 could reduce CYP2C19 protein level in human primary hepatocytes [17]. Also, miR-34a, miR-130b, miR-185 displayed a negative correlation with *CYP2C9* mRNA level in human liver tissue [10]. Recently, in silico analysis indicated *CYP2C19* can be targeted by miR-29a-3p [19]. In addition, an inverse correlation was found between miR-29a-3p and CYP2C19 mRNA or protein expression in HepaRG cells and human liver tissue samples [19]. CYP2E1 catalyzes the oxidation of many solvents and other small organic molecules. A potential miR-378 binding site was identified in the 3’-UTR of *CYP2E1* mRNA. In HEK293 cell lines, miR-378 overexpression can decrease CYP2E1 protein expression and chlorzoxazone 6-hydroxylation activity [20]. In addition, miR-378 expression correlates inversely with protein level and translational efficiency of CYP2E1 in human liver [20]. A recent luciferase assay suggested that miR-132 and miR-212 can directly target *CYP2E1* 3’-UTR, which may play a role in insulin-induced inhibition of CYP2E1 expression in primary cultured rat hepatocytes [21]. These findings helped to understand post-transcriptional regulation of *CYP2E1*. Also, the negative correlation between miR-10a, let-7g, and miR-200c and *CYP2E1* mRNA level was reported [10].

CYP2J2 has been found to catalyze epoxidation and hydroxylation of polyunsaturated fatty acids. More recently, let-7b was shown to repress the expression of epoxygenase CYP2J2 directly, and let-7b downregulation is associated with increased CYP2J2 expression in lung cancer [22].

CYP3A4 metabolizes more than 50% of therapeutic drugs. The variability of CYP3A4 expression may contribute to inter-individual difference in drug response. MiR-27b was identified to inhibit CYP3A4 mRNA/protein expression and miR-27b overexpression in PANC1 cells decreases the sensitivity to cyclophosphamide [23]. In addition, miR-577, miR-1, miR-532-3p, and miR-627 were observed to repress CYP3A4 protein expression in cultured HEK293T cells, and downregulated the translation efficiency of *CYP3A4* mRNA in human livers [24]. Negative correlations between levels of miRNAs including miR-155, miR-454, miR-582-5p, let-7f-1*, miR-181d, and miR-500 and hepatic CYP3A activity were observed in cirrhotic livers [25]. A recent study demonstrated that miR-27a could negatively regulate CYP3A4 mRNA and protein level in 26 human liver samples [26]. These results may reveal a difference in post-transcriptional regulation of CYPs by miRNA in cancer cells and normal tissues, but also in in vitro versus in vivo.

CYP7A1 is important for the regulation of bile acid synthesis in the liver. Overexpression of miR-122a and miR-422a inhibited, whereas their inhibitors promoted *CYP7A1* mRNA expression in human hepatocytes [27]. And luciferase reporter assay identified the binding sites of miR-122a and miR-422a in the *CYP7A1* 3’UTR.

CYP24A1 is a key enzyme in the inactivation of calcitriol, which exerts antiproliferative effects in cancer cells by binding to the vitamin D receptor. It has been reported that CYP24A1 may be a candidate oncogene and a potential prognostic biomarker for cancer [28]. A potential miR-125b recognition element was identified in the 3’-UTR of *CYP24A1* and *VDR* [29,30]. CYP24A1 protein expression was increased in breast cancer tissues, which could be explained by decreased miR-125b expression [30]. As VDR can regulate transcription of *CYP24A1*, miR-125b is supposed to regulate CYP24A1 expression directly and indirectly.

### 3.2. Direct Repression of Drug Transporters by miRNAs

Drug transporters are a group of membrane proteins that are responsible for the transportation of drugs into and out of cells. Drug transporters can be divided into uptake and efflux transporters. Uptake transporters are involved in the uptake of endogenous and exogenous substances. The soluble carrier (SLC) family are major types of uptake transporters. Efflux transporters are primary active transporters, belonging to the energy-dependent ATP-binding cassette (ABC) superfamily. Overexpression of one or more ABC transporters accounts for decreased intracellular accumulation of chemotherapeutic drugs in cancer cells and thus potentiate multidrug resistance (MDR) [31]. The cellular mechanisms of MDR include decreased drug uptake, increased drug efflux, activation of detoxifying systems, activation of DNA repair mechanisms, evasion of drug-induced apoptosis, etc. Currently, the most widely studied cellular mechanisms of tumor resistance are those associated with ABC transporter-mediated drug efflux [32]. Interests in miRNA-mediated modification of the expression of drug transporters focused on cancer MDR is increasing.

Multidrug resistance protein 1 (MDR1/ABCB1/P-pg) is involved in efflux of numerous drugs including antibiotics, anticancer drugs, and antiviral agents. Boyerinas et al., showed that let-7g overexpression in MDR1 positive ADR-RES cells led to a reduced P-pg expression [33]. An inverse correlation was observed between let-7g level and P-pg expression in ovarian cancer patients [33]. The expression of P-gp and *MDR1* mRNA can be upregulated by miR-27a and miR-451, which were observed to be higher expressed in the MDR cancer cell lines as compared with their parental lines [34]. In addition, miR-27a and miR-451 antagomir can decrease the expression of P-gp and *MDR1* mRNA and increase vinblastine sensitivity in MDR ovarian cancer cells [34]. MiR-27a was further identified to repress P-gp and *MDR1* mRNA by targeting HIPK2 in cancer cell lines A2780 and A2780/Taxol [35]. Transfection of miR-451 mimics increases sensitivity to doxorubicin in doxorubicin-resistant MCF-7 cells and irinotecan in colon carcinoma cells by binding to the *MDR1* 3’-UTR [36]. These findings help to understand why patients with lower miR-451 level respond worse to irinotecan-based therapy [36]. Furthermore, overexpression of miR-298 and miR-223 was reported to downregulate P-gp expression, and increase doxorubicin sensitivity in doxorubicin-resistant breast cancer cells and HCC cells [37,38]. Recently, miR-508-5p was reported to suppress expression of P-gp and *MDR1* mRNA by directly targeting the 3’-UTR of *ABCB1.* MiR-508-5p overexpression can sensitize tumours to chemotherapy in vivo in gastric cancer [39]. Another study revealed that miR-145 could regulate the expression and function of P-gp in intestinal epithelial cells [40].

ABCB9 is a brain and spinal cord lysosome-associated transporter. It has shown that miR-31 could inhibit cisplatin-induced apoptosis via regulating ABCB9 expression in non-small cell lung cancer cells [41]. And luciferase assay confirmed that *ABCB9* is a direct target of miR-31 [41].

Multidrug resistance-associated protein 1 (MRP1/ABCC1) mediated the active efflux of glucuronide, glutathione, and sulfate conjugates [42]. Liang and colleagues found that miR-326 expression in VP-16-resistant MCF-7 cells (MCF-7/VP) was higher than that in MCF-7 cells [43]. Furthermore, miR-326 overexpression decreases ABCC1 mRNA and protein expression by direct targeting and sensitizing MCF-7/VP cells to VP-16 and doxorubicin [43]. To identify differentially expressed miRNAs in drug sensitive and resistant small cell lung cancer cell lines that might underlie MDR, Guo et al., observed that miR-134 downregulated and ABCC1 upregulated in drug resistant cells, and miR-134 was a causal factor for downregulation of ABCC1 [44]. The liver-specific miR-122 was also reported to render adriamycin and vincristine sensitivity through inhibiting ABCB1 and ABCC1 expression in HCC [45]. In human pancreatic carcinoma PANC-1 cells, ABCC1 expression is sharply reduced by miR-1291 transfection, while miR-1291 antagomir exhibited the opposite effect [46]. The miR-1291-directed downregulation of ABCC1 sensitized the PANC-1 cells to doxorubicin [46]. Recently, miR-7 modulates chemoresistance of small cell lung cancer through modulating protein expression of ABCC1 [47].

ABCC2 plays an important role in resistance to platinum-based chemotherapy. Haenisch and colleagues found that miR-379 impedes ABCC2 protein expression by directly targeting 3’-UTR of *ABCC2* in HepG2 cells [48,49]. MiR-297 was proved to reduce ABCC2 protein expression in MDR colorectal carcinoma cells and sensitize these cells to anticancer drugs including oxaliplatin, vincristine, doxorubicin, 5-fluorouracil and mitomycin C [50]. A recent study demonstrated that let-7c sensitizes acquired cisplatin-resistant A549 cells by targeting *ABCC2* [51].

Multidrug resistance-associated protein 4 (MRP4/ABCC4) is involved in the transport of endogenous and xenobiotic organic anionic compounds. Borel and colleagues found that miR-125a/b could regulate *ABCC4* mRNA expression by direct targeting 19 paired HCC tissue [52]. More recently, miR-124a and miR-506 were reported to decrease protein levels and function of ABCC4 in HEK293T/17 cells [53]. Furthermore, a negative correlation between miR-124a and miR-506 expression and MRP4 protein expression was observed in 26 human kidney samples [53].

Borel et al., observed an inverse correlation between ABC transporters and miRNA expression levels in the HCC tissues, and 13 miRNAs were confirmed to target *ABCA1*, *ABCC1*, *ABCC5*, *ABCC10* and *ABCE1* directly [52]. The expression of miR-128 was reduced accompanying by ABCC5 overexpression in chemoresistant breast tumor-initiating cells, which may partially explain why reduced miR-128 expression was associated with chemotherapeutic resistance and poor survival in breast cancer [54].

Breast cancer resistance protein (BCRP/ABCG2) was initially discovered in multidrug resistant breast cancer cell lines, where it confers resistance to several chemotherapeutic agents. Substrates for ABCG2 include mitoxantrone, topotecan, irinotecan, methotrexate, and tyrosine kinase inhibitors such as imatinib and gefitinib [55]. ABCG2 was supposed to contribute to xenobiotica protection for stem cells and underlies the ability of cancer cells to regenerate post-chemotherapy [56]. MiR-328 and miR-519c were observed to suppress ABCG2 protein expression by targeting the *ABCG2* 3’-UTR [57,58,59]. Subsequently, Li and colleagues showed that miR-519c downregulates ABCG2 protein expression with accelerating *ABCG2* mRNA degradation and overexpression of miR-519c or miR-328 in MCF-7 cells could increase intracellular mitoxantrone accumulation, which is probably due to a decreased ABCG2 protein expression [60]. In chronic myeloid leukemia K-562 cells, short-term imatinib treatment induced ABCG2 expression and decreased miR-212 expression, while anti-miR-212 upregulated ABCG2 protein expression by direct targeting *ABCG2* 3’-UTR [61]. Two separate groups have demonstrated that miR-520h can regulate ABCG2 expression by direct inhibition [62,63]. In addition, miR-181a and miR-487a can sensitize mitoxantone-resistant breast cancer cells to chemotherapeutic agents by targeting *ABCG2* [64,65].

SLC6A4, also known as serotonin transporter (SERT), is involved in serotonin reuptake. It is the pharmacological target of selective serotonin reuptake inhibitor antidepressants [66]. Data from Baudry and colleagues suggested that miR-16 could target *SLC6A4* in neuronal 1C11 cell line [66]. In mice, chronic fluoxetine treatment can increase miR-16 levels in serotonergic raphe nuclei, which subsequently downregulate SLC6A4 expression [66]. The expression of miR-16 was negatively correlated with SLC6A4 expression in mouse and miR-16 overexpression lead to decrease of SLC6A4 in human alveolar epithelial cells [67]. Furthermore, miR-15a and miR-16 could regulate SLC6A4 expression in human placental choriocarcinoma and rat brain raphe cells [68].

SLC7A5 is also known as L-type amino acid transporter 1 (LAT1). MiR-126 was reported to inhibit proliferation of small cell lung cancer cells by targeting *SLC7A5* [69]. Drayton and colleagues found that miR-27a suppresses protein expression of cystine/glutamate transporter SLC7A11 in cisplatin-resistant bladder cancer. Bladder cancer with low miR-27a or high SLC7A11 expression exhibited poorer clinical outcomes [70].

Data from Gillen and colleagues has shown that miR-384, miR-494, and miR-1246 could directly repress mRNA expression of Na-K-Cl co-transporter SLC12A2 in epithelial cells [71]. The peptide transporter 1 (PEPT1/SLC15A1) is involved in intestinal absorption of small peptides and a variety of peptidemimetic drugs such as β-lactam antibiotics. MiR-92b was reported to directly downregulate mRNA and protein levels of SLC15A1 and thus reduces SLC15A1-mediated drug transport activity [72]. The H-linked monocarboxylate transporter isoform 1 (MCT1/SLC16A1) is involved in the transportation of metabolically important monocarboxylates such as lactate, pyruvate, acetate and ketone bodies. Evidence shows that miR-124 could negatively regulate SLC16A1 expression at both mRNA and protein levels [73,74].

Up to date, most studies regarding miRNA-mediated regulation of drug transporters mainly focused on MDR in cancer cells [8]. Targeting specific miRNAs of the drug-resistant network is promising in overcoming drug resistance in cancer therapy. The miRNA-mediated modification of drug transporters and the clinical relevance requires further investigation.

### 3.3. Indirect Regulation of DMEs and Drug Transporters by miRNAs

The expression of genes involved in the disposition of drugs is largely regulated by transcription factors belonging to the xenobiotic-sensing nuclear receptors family such as pregnane X receptor (PXR), constitutive androstane receptor (CAR), and hepatocyte nuclear factor 4 alpha (HNF4α). Nuclear receptors are important in regulation of both the constitutive and inducible expression of DMEs and transporters. MiRNA-mediated regulation of the expression of nuclear receptors becomes potential mechanism for controlling DMEs and transporters.

HNF4α is a key transcription factor that regulates the expression of numerous DMEs and drug transporters such as CYPs, UDP-glucuronosyltransferases (UGTs), ABC transporters, OATs, and OATPs [75]. It was found that miR-24 suppresses *HNF4α* mRNA expression mainly through mediating its mRNA degradation, while miR-34a acts through direct translational repression [76]. Overexpression of miR-24 and miR-34a resulted in decreased HNF4α protein level and its subsequent targets such as CYP7A1 and CYP8B1 in HepG2 cells [76]. Further study showed that HNF4α protein levels were reduced by transfection of miR-24 and miR-629 mimics in HepG2 cells [77]. Also, miR-34a and miR-449a could downregulate HNF4α protein expression and *PXR* mRNA levels [78]. In addition, overexpression of miR-34a, miR-34c-5p and miR-449a lead to decrease in the protein levels and binding activity of HNF4α [79].

PXR is an important xenoreceptor regulating the inducible expression of a variety of transporters and DMEs, including CYP3A4. It was reported that miR-148 directly inhibits PXR protein expression and negatively regulates the translational efficiency of *PXR* in 25 human liver samples [80]. MiR-148a could also inhibit the constitutive/inducible *CYP3A4* mRNA expression in a PXR-dependent manner [80]. However, subsequent study failed to observe the correlation between miR-148 and protein and mRNA level of PXR or CYP3A4 in human liver samples [10,81].

Vitamin D receptor (VDR) is a nuclear hormone receptor, which functions as transcription factors by binding to vitamin D response element in the promoters of the target genes, such as *CYP3A4* [82]. MiR-27b was found to indirectly regulate CYP3A4 by indirect targeting the *VDR* 3’-UTR and by direct targeting [23]. Furthermore, miR-125b could decrease VDR protein level in MCF-7 cells by binding to recognition element within *VDR* 3’UTR [30]. Hence, miR-125b may be supposed to indirectly influence CYP3A4 via VDR mediated posttranscriptional regulation.

Glucocorticoids such as cortisol exhibit profound role in neuronal development, immunity, and metabolism by binding to the glucocorticoid receptor (GR) [83]. It was found that GR could regulate the expression of CYP2C and CYP3A4 [84]. Vreugdenhil et al., demonstrated that miR-18 and miR-124a decreased GR-mediated events in addition to reducing GR protein levels in neuronal tissues [85]. However, the miRNA-mediated posttranscriptional regulation of GR exhibits no correlation with xenobiotic biotransformation.

Estrogen receptor 1 (ESR1), an estrogen-activated nuclear receptors, is involved in regulation of CYP1B1 expression. Data form Adams and colleagues demonstrated that miR-206 suppresses ESR1 mRNA and protein level in breast cancer cell lines [86]. MiR-221 and miR-222 suppress ESR1 protein level in MCF-7 and T47D cells [87]. Overexpression of miR-221 or miR-222 resulted in the breast cancer cell line becoming resistant to tamoxifen [87]. Also, miR-22 directly inhibited ESR1 mRNA and protein expression in breast cancer cell lines and clinical biopsies [88,89]. In addition, ESR1 was a direct target of miR-130a in HepG2.2.15 human HCC cells [90].

Peroxisome proliferator activated receptor alpha (PPARα) is a nuclear hormone receptor family transcription factor, which is involved in regulation of DME and tumor progression. MiR-10b was proven to inhibit PPARα protein expression in steatotic L02 cells [91]. Also, miR-21 and miR-27b could regulate PPARα protein level in Huh7 cells [92]. However, these miRNAs exhibited no effect on *PPARα* mRNA levels. Tong et al., showed that miR-506 overexpression in a colon cancer cell could inhibit PPARα expression, which resulted in hydroxycamptothecin resistance [93]. Another nuclear receptor liver X receptor α (LXRα) has been found to be suppressed by miR-613 in HepG2 cells [94,95]. A recent study revealed that miR-206 inhibits LXRα protein expression and promotes LXR-mediated cholesterol efflux in macrophages [96].

### 3.4. MiRNAs Mediate Drug-Drug Interactions in Pharmacokinetics

Xenobiotic agent induced dysregulation of miRNAs, which regulates the expression of DEMs and drug transports, may result in considerable alterations in the pharmacokinetic profile of a concomitant drug [97]. Rodrigues et al., revealed that the expression of several miRNAs (miR-27a, miR-124a, miR-148a, and miR-451) in MCF-7, Caco-2, SH-SY5Y and BE(2)-M17 cell lines can be influenced by exposure to 19 xenobiotic drugs, including methadone, dexamethasone, gemcitabine, imatinib, and mitoxantrone [98]. In this respect, the upregulation of CYP3A4 and ABCB1 by dexamethasone may involve the suppression of dexamethasone on miR-27b, miR-148a and miR-451 [98], which could target 3’-UTR of *CYP3A4* and *ABCB1*. Moreover, bilobalide led to a decreased level of miR-148a [98], which could directly inhibit PXR protein expression. Neuronal miR-124a was reduced by treatment with psychoactive drugs (cocaine, methadone and fluoxetine) [98], which may provide increased understanding of neuroplasticity. Dysregulated expression of miR-10a, miR-146a, miR-200b, miR-200c, miR-221/222, and miR-345 induced by drugs could result in chemoresistance to cisplatin in MCF-7 breast cancer cells [99].

Rifampicin is a well-known drug inducer that activates PXR/RXR. The induction of *MDR1* and *CYP2B6* mRNA by rifampicin are attenuated by miR-148a overexpression in LS180 cells [80]. Rifampicin could downregulate ABCC2 protein expression by increasing miR-379 expression in HepG2 cells [48]. In primarily cultured hepatocytes, rifampicin upregulated and downregulated the expression of a set of miRNAs, and some of the mRNA/miRNA pairs were inversely associated [100,101]. Hence, delineation of the influence of xenobiotic drugs on miRNA profile might present a mechanism of altered gene expression underlying drug disposition and drug-drug interaction.

## 4. MiRSNPs Modify Cancer Chemotherapy Response and Survival

**Figure 2:**
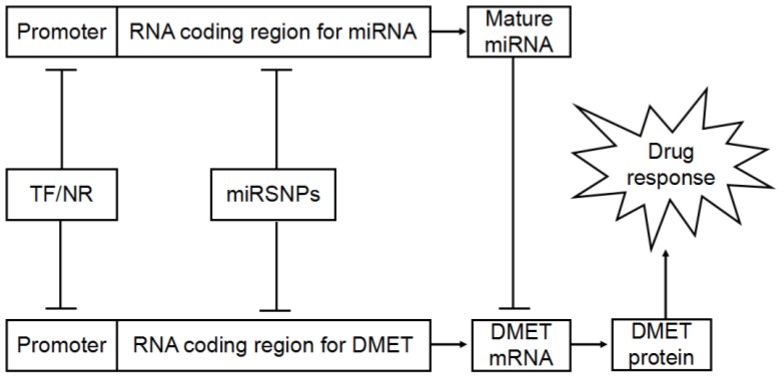
Interplay between miRNA-mediated posttranscriptional regulation of drug disposition related genes expression and drug response. DMET: drug-metabolizing enzymes and transporters; TF: transcription factors; NR: nuclear receptors.

A class of functional polymorphisms termed miRNA polymorphisms or miRSNPs are reported to be a new player in miRNA-mediated gene regulation (Figure 2). MiRSNPs refer to polymorphisms present at or near miRNA binding sites of functional genes as well as in genes involved in miRNA biogenesis and in pri-, pre- and mature miRNA sequences. A growing number of miRNAs related causative SNPs were identified [102]. Disease susceptibility associated miRSNPs have attracted growing interests [103]. However, knowledge about the pharmacogenomic significance of the miRSNPs is scarce. Most interest in miRSNPs is focused on cancer chemotherapy resistance and survival. Numerous miRSNPs associated with chemotherapy response and clinical outcomes are identified (Table 2).

### 4.1. MiRNA Target Site Polymorphisms

Lung cancer (LC) is the leading cause of cancer-related deaths worldwide. Non-small cell lung cancer (NSCLC) accounts for 80% of all lung cancer cases and less than 15% of patients with NSCLC survive beyond 5 years [104]. Thus, identification of specific prognostic biomarkers may improve the medical care of patients with NSCLC. Increased SET8 expression was observed in various types of tumor, including LC. *SET8* rs2240688 T > C, a polymorphism within miR-502 binding site, was reported to be associated with increased overall survival (OS) or reduced risk of death in small-cell lung cancer (SCLC) [105] and NSCLC [106]. SET8 modifies cancer prognosis by altering its expression, which could be suppressed by miR-502. Another miRSNP, rs2240688 A > C, within the 3′-UTR of *CD133* was associated with favorable prognosis [107]. Functional assays revealed that rs2240688 C allele creates a new binding site for miR-135a/b and thus reduced *CD133* mRNA level [107]. CD133 was overexpressed in several human cancer tissues and was associated with poor prognosis [107]. In another study on NSCLC, *KTR81* rs3660 polymorphism within potential miR-17 target site was associated with increased time to recurrence of NSCLC [108]. In addition, evidence revealed that *FAS* rs2234978 common allele was associated with longer OS of NSCLC [109]. In the same study, the G allele of *FZD4* rs713065 was associated with longer OS of early NSCLC [109]. Luciferase reporter assays showed that minor allele of rs2234978 and rs713065 created binding site for miR-651 and miR-204 [109,110].

Colorectal cancer (CRC) is the second most common malignancy and the fourth-leading cause of cancer death worldwide. Evidences revealed that miRSNPs may represent prognosis markers of CRC. The variant allele of LCS6 polymorphism (rs61764370 T > G) in the binding site of let-7 to *KRAS* 3’-UTR was reported to be associated with reduced OS and progression-free survival (PFS) in metastatic colorectal cancer (mCRC) [111], and nonresponse to anti-EGFR-based treatment in *KRAS* and *BRAF* wild-type mCRC patients [112]. However, the conflict results were also observed, in which LCS6 common allele exhibited association with shorter PFS/OS or no effect on them [113,114]. The contradictions may be explained by small sample size and the different inclusion criteria for the mutations in each study. Further clinical studies are needed to increase the accuracy in predicting cetuximab responsiveness based on the LCS6 polymorphism.

Ovarian cancer (OC) is the most lethal gynecological malignancy and the 5-year survival rate is less than 30% [115]. A recent study revealed that variant allele of *MDM4* 3’-UTR polymorphism rs4245739 abrogates the miR-191 target site and results in increased MDM4 expression, which was associated with increased risk for recurrence, accelerated tumor progression, and chemotherapy resistance in ovarian carcinoma [115]. In a study on 417 Caucasian patients with OC, another potential miR-409-3p recognition site polymorphism, *MDM4* rs10900596 G > A, was related to an improved treatment response in ovarian cancer [116]. In the same study, the rs1425486 variant allele disrupts *PDGFC* pairing with miR-425, inhibits miR-425 targeting, and results in higher PDGFC expression and worse OS [116]. Moreover, the variant allele of *KRAS* rs10771184 within potential miR-544 binding site was associated with better treatment response and increased OS in OC [116].

Several miRSNPs exhibited association with survival of other tumors. The rs2240688 CC genotype was identified to be associated with reduced SET8 protein levels and longer postoperative OS in Asian patients with HCC [117]. The results suggest that SET8 modifies cancer prognosis by altering its expression, which could be suppressed by miR-502. Evidence indicates that *KTR81* rs3660 polymorphism within potential miR-17 target site was associated with toxicity or survival of Hodgkin lymphoma [118] and multiple myeloma [119]. Furthermore, the rs1045385 C allele of *AP-2α* rs1045385 polymorphism was insensitive to miR-200b/200c/429 induced repression of AP-2α expression and increases cisplatin sensitivity in endometrial cancer cell line HEC-1A cells [120]. In addition, the *CDON* rs3737336 polymorphism located in the miR-181c/miR-5007 binding site was associated with reduced PFS of prostate cancer [121,122].

### 4.2. Polymorphisms in miRNA Biogenesis Gene

Numerous miRSNPs associated with NSCLC chemotherapy toxicity or survival were identified. *MiR-196a2* rs11614913 C > T polymorphism could alter mature miRNA expression and function. Rs11614913 CC homozygotes exhibited higher occurrence of overall toxicity in response to gemcitabine or cisplatin [123]. Also, rs11614913 T allele was associated with better OS and disease-free survival (DFS) [124]. In the same study, the minor allele carriers of *miR-149* rs2292832 exhibited better OS and DFS [124]. Recent evidence indicated that G allele of *pre-miR-27a* rs895819 was associated with decreased response rate to platinum-based chemotherapy, reduced OS and increased risk of death in NSCLC [125]. In another study on 452 early-stage and 526 late-stage NSCLC patients, minor allele of *miR-5197* rs2042253 was associated with increased OS [126]. Recently, *DROSHA* rs6886834 minor allele was associated with shorter median recurrence-free time [109]. Furthermore, *XPO5* 3’-UTR polymorphism rs11077 was identified as predictor for recurrence of NSCLC [108] and survival of SCLC [127].

There are several miRSNPs as markers of clinical outcome of CRC. Boni et al., found two polymorphisms were associated with clinical outcome in metastatic colon cancer patients treated with 5-fluorouracil and irinotecan [128]. The rare homozygous genotype of *pri-miR26a-1* rs7372209 exhibited poorer response and reduced time to progression [128]. And the minor allele of another polymorphism *pri-miR-100* rs1834306 was associated with increased time to progression [128]. The minor allele of *miR-608* rs4919510 was associated with shorter recurrence-free survival (RFS) and the minor allele homozygotes in *miR-219-1* rs213210 exhibited increased RFS of colorectal adenocarcinoma [129]. Recently, major allele in *miR-219-1* rs213210 was reported to be associated with worse OS [130]. In the same study, carriers of minor allele in *miR-608* rs4919510 exhibited longer event-free survival (EFS) [130].

Evidences have shown that miRSNPs may be associated with survival in urinary system cancers. Two miRSNPs, *KIF3C* rs6728684 and *IFI30* rs1045747, were associated with reduced PFS in prostate cancer treated with androgen-deprivation therapy [121]. The variant allele of *DDX20* rs197412 conferred a decreased risk of recurrence and the variant homozygous genotype of *DGCR8* rs2073778 was associated with increased risk of progression in non-muscle-invasive bladder cancer [131].

MiRSNPs were also identified as prognostic markers for other cancers. *XPO5* rs11077 polymorphism exhibited association with OS and PFS of multiple myeloma [119], chemotherapy toxicity and OS of Hodgkin lymphoma [118], and survival of HCC [132]. In addition, the G allele of rs17408716 within *RNASEN* was associated with better treatment response and longer OS of OC [116].

### 4.3. CNV-miRNA: Possible Causative Variations Affecting Drug Response

Copy number variations (CNVs) refer to segments of genomic DNA that show variable numbers of copies in the genome due to deletions or duplications. CNVs affect gene expression in a copy number-dependent manner and account for about 18% detected genetic variation in gene expression [133]. CNVs in miRNA genes (CNV-miRNAs) affect binding and regulation of miRNA target genes [134]. Evidences have revealed that CNV-miRNAs were involved in a broad range of phenotypes, including male fertility [135], multiple sclerosis [136], and autism [137]. More recently, 209 CNV-miRNAs were identified in CNV regions, and 4 miRNAs (miR-1268, miR-1233, miR-1972, and miR-384) were located in polymorphic CNV regions [138]. These miRNA-CNVs include deletions (miR-384 and miR-1324), duplications (miR-1972 and miR-1977), and multiple duplications (miR-1233 and miR-1268) [138]. Significance of these miRNA-CNVs remains to be explored. Therefore, there is still a long way to go before the implication of these miR-CNVs into pharmacogenomics and personalized medicine.

## 5. MiRNAs and Ethnic Difference in Drug Response

Evidences revealed that polymorphisms in miRNAs have significantly different frequencies among various populations [139,140]. And these polymorphisms may lead to severe defects to functions of miRNAs and then possible ethnic difference in drug response. Previous studies have demonstrated that 17%–30% of genes are differentially expressed among different ethnic populations [140,141] and miRNAs are also expressed in a population-specific manner [141,142]. The differentially expressed genes and miRNAs may be another basis of ethnic difference in drug response.

A total of 1899 SNPs in 961 reported pre-miRNAs were identified [139]. Among them, some SNPs exhibited significantly different frequencies between various populations in the HapMap and 1000 Genome Projects [139]. Recently, Rawlings-Goss et al., identified 31 miRSNPs that were globally population-differentiated in frequency between African and non-African populations [140]. Moreover, miR-202, a potential breast cancer biomarker, exhibited significantly high allele frequency differentiation at rs12355840 polymorphism, which influences miRNA expression in vivo and breast cancer mortality [140]. The role of these SNPs in the inter-ethnic difference in drug response needs further investigation.

Previous studies identified many miRNAs that exhibit ethnic difference in expression and prognostic significance for cancer. A total of 33 differentially expressed miRNAs between CEU and YRI were identified in HapMap lymphoblastoid cell lines [142]. More than 55% of the differentially expressed miRNAs were inversely correlated with an mRNA expression phenotype in each population samples, and 21 of these miRNAs correlated with cellular sensitivity to at least one of the chemotherapeutic agents in carboplatin, daunorubicin, and cytarabine [142]. Moreover, a large number of SNPs exhibiting different allele frequencies affected the expression of the differentially expressed miRNAs [142]. High miR-181b expression in cancer tissue correlated with poor survival of black rather than white patients with CRC [143]. The expression levels of plasma miR-375 are ethnically different in diabetes of Han and Kazak populations [144]. Furthermore, higher plasma miR-144 expression was significantly associated with diabetes in Swedes, but not in Iraqis [145]. MiR-182, miR-152, miR-204, miR-222 and miR-202 exhibited differential expression in colon cancer between African and Caucasian Americans [146]. Notably, miR-182 was increased and two potential miR-182 targets (*FOXO1* and *FOXO3A*) were decreased in African Americans tumors, which may contribute to decreased colon cancer survival in African Americans [146].

## 6. Conclusions

MiRNAs emerge as a new player in epigenetic regulation of genes involved in drug response. MiRSNPs are recently found to be associated with progression and prognosis of different types of cancers. Detection of miRNAs and miRSNPs holds promise in the field of miRNA pharmacogenomics for individualized medicine. The challenge for achieving individualized drug therapy is manyfold. Establishing the relation of miRNAs and miRSNPs to drug response phenotypes may not be straightforward. Factors affecting drug response are multifold and complex. At present, individualized drug therapy may be achieved when these factor are simple and well defined. Several important achievements, such as warfarin therapy based on *VKORC1* and *CYP2C9* genotypes, have been achieved. Large scale, prospective clinical trials are needed to reveal causal associations between genetic as well as epigenetic variations and drug response. It is safe to say that more pharmacoepigenomic biomarker of drug response and stronger supporting clinical research evidence are expected to surface in the coming years.

## Figures and Tables

**Table 1:** MiRNAs reported to regulate drug metabolism related genes.

Function	Gene	Involved miRNA	Identification Methods	References
DMEs	CYP1A1	miR-18b, -20b	Correlation, mRNA expression	[9]
		miR-892a	Reporter assay, mRNA/protein expression, functional assay	[11]
	CYP2A3	miR-126* ^a^	mRNA/protein expression	[13]
	CYP1B1	miR-27b	Correlation, Reporter assays, protein expression, functional assay	[14]
	CYP2C8	miR-103, -107	Reporter assay, mRNA/protein expression, correlation	[17]
	CYP2C9	miR-128-3p	EMSA, reporter assay, mRNA/protein expression, correlation	[18]
	CYP2C19	miR-103, -107	Protein expression	[17]
		miR-29a-3p	EMSA, mRNA/protein expression, correlation	[19]
	CYP2E1	miR-378	Reporter assay, mRNA/protein expression, correlation, functional assay	[20]
		miR-132, -212	Reporter assay, mRNA expression	[21]
	CYP2J2	let-7b	Reporter assay, protein expression, functional assay	[22]
	CYP3A4	miR-27b	Reporter assay, mRNA/protein expression, functional assay	[23]
		miR-1, -532-3p, -577, -627	Reporter assay, protein expression, correlation	[24]
		miR-27a	Reporter assay, mRNA/protein expression, correlation	[26]
	CYP7A1	miR-122a, -422a ^a^	Reporter assay, mRNA expression	[27]
	CYP24A1	miR-125b	Reporter assay, mRNA/protein expression, functional assay	[29]
Transporters	ABCB1/MDR1	let-7g	mRNA/protein expression, correlation, functional assay	[33]
		miR-27a	mRNA/protein expression, functional assay	[34,35]
		miR-451 ^a^	mRNA/protein expression, functional assay	[34,36]
		miR-298 ^a^	Reporter assay, protein expression, functional assay	[37]
		miR-223	Reporter assay, mRNA/protein expression, functional assay	[38]
		miR-508-5p	Reporter assay, mRNA/protein expression, functional assay	[39]
		miR-145	Reporter assay, protein expression, functional assay	[40]
	ABCB9	miR-31	Reporter assay, mRNA/protein expression, functional assay	[41]
	ABCC1/MRP1	miR-326	Reporter assay, mRNA/protein expression, functional assays	[43]
		miR-134	mRNA/protein expression	[44]
		miR-122	mRNA/protein expression, functional assay	[45]
		miR-1291 ^a^	Reporter assay, mRNA/protein expression, and functional assays	[46]
		miR-7	Reporter assay, mRNA/protein expression, correlation	[47]
	ABCC2/MRP2	miR-379	Reporter assay, mRNA/protein expression, functional assays	[48,49]
		miR-297 ^a^	Reporter assay, mRNA/protein expression, functional assays	[50]
		let-7c	Reporter assay, mRNA/protein expression, functional assays	[51]
	ABCC4/MRP4	miR-125a/b	Reporter assay, mRNA expression, correlation	[52]
		miR-124a, -506 ^a^	Reporter assay, mRNA/protein expression, correlation, functional assay	[53]
	ABCC5/MRP5	miR-128	Reporter assay, protein expression, functional assay	[54]
	ABCG2/BCRP	miR-328	Reporter assay, mRNA/protein expression, functional assays	[57,58,59]
		miR-519c	Reporter assay, mRNA/protein expression, functional assays	[60]
		miR-212	Reporter assay, protein expression	[61]
		miR-520h	Reporter assay, mRNA/protein expression	[62,63]
		miR-181a	Reporter assay, mRNA/protein expression, functional assays	[64]
		miR-487a	Reporter assay, mRNA/protein expression, functional assays	[65]
	SLC6A4/SERT	miR-16	Reporter assay, mRNA/protein expression, functional assays	[66,67,68]
		miR-15a	Reporter assay, protein expression	[68]
	SLC7A5/LAT1	miR-126	Reporter assay, mRNA/protein expression, correlation	[69]
	SLC7A11	miR-27a	Reporter assay, mRNA/protein expression, functional assays	[70]
	SLC12A2	miR-384 ^a^, -494, -1246 ^a^	Reporter assay, mRNA expression	[71]
	SLC15A1/PEPT1	miR-92b	Reporter assay, mRNA/protein expression, correlation, functional assays	[72]
	SLC16A1/MCT1	miR-124	Reporter assay, mRNA/protein expression, functional assays	[73,74]
Nuclear Receptor	HNF4α	miR-24, -34a	Reporter assay, mRNA/protein expression, functional assays	[76,77]
		miR-34a, -34c-5p, -449a	Reporter assay, mRNA/protein expression, functional assays	[78,79]
	PXR	miR-148a	Reporter assay, protein expression	[80]
	VDR	miR-27b	Reporter assay, mRNA/protein expression	[23]
		miR-125b	Reporter assay, EMSA, protein expression	[30]
	GR	miR-18, -124a	Reporter assay, protein expression, functional assays	[85]
	ESR1	miR-206	Reporter assay, mRNA/protein expression	[86]
		miR-221, -222	Reporter assay, protein expression, functional assays	[87]
		miR-22	Reporter assay, mRNA/protein expression	[88,89]
		miR-130a	Reporter assay, mRNA/protein expression	[90]
	PPARα	miR-10b, -21, -27b	Reporter assay, mRNA/protein expression, correlation	[91,92]
		miR-506	Reporter assay, mRNA/protein expression, functional assays	[93]
	LXRα	miR-613 ^a^	EMSA, reporter assay, mRNA/protein expression, functional assays	[94,95]
		miR-206	Reporter assay, mRNA/protein expression, functional assays	[96]

^a^ MiRNAs that are not listed as bona fide miRNAs in MirGeneDB [5]. EMSA: electrophoresis mobility shift assay.

**Table 2:** MiRSNPs as biomarkers of chemotherapy response and survival.

Gene	MirSNPs	Involved miRNAs	Cancer Types	Association with Variant Allele	References
SET8	rs16917496 T > C	miR-502	SCLC	Increased OS	[105]
			NSCLC	Increased OS and reduced risk of death	[106]
CD133	rs2240688 A > C	miR-135a/b	LC	Increased OS	[107]
KRT81	rs3660 C > G	miR-17	NSCLC	Increased time to recurrence	[108]
FAS	rs2234978 A > G	miR-651	NSCLC	Reduced OS	[109]
FZD4	rs713065 A > G	miR-204	NSCLC	Increased OS	[109,110]
KRAS	rs61764370 T > G	let-7	CRC	Poor response to cetuximab-irinotecan therapy and reduced OS/PFS	[111]
			CRC	Resistance to anti-EGFR-based therapy	[112]
			CRC	Improved OS/PFS	[113,114]
MDM4	rs4245739 C > A	miR-191	OC	Delayed progression and tumor related death	[115]
	rs10900596 G > A	miR-409-3p ^a^	OC	Better treatment response	[116]
PDGFC	rs1425486 G > A	miR-425	OC	Poor treatment response and reduced OS	[116]
KRAS	rs10771184 T > A	miR-544	OC	Better treatment response and increased OS	[116]
SET8	rs16917496 T > C	miR-502	HCC	Increased OS	[117]
KRT81	rs3660 C > G	miR-17	HL	Increased risk of neurological toxicity	[118]
KRT81	rs3660 C > G	miR-17	MM	Increased OS	[119]
AP-2α	rs1045385 A > C	miR-200b, -200c, -429	BC	Increased cisplatin sensitivity	[120]
CDON	rs3737336 T > C	miR-181c, -5007 ^a^	PC	Recreased PFS	[121,122]
miR-196a-2	rs11614913 C > T		NSCLC	Decreased risk of overall toxicity and increased OS/DFS	[123,124]
miR-149	rs2292832 T > C		NSCLC	Increased OS/DFS	[124]
pre-miR-27a	rs895819 A > G		NSCLC	Poor treatment response and reduced OS	[125]
miR-5197 ^a^	rs2042253 T > C		NSCLC	Increased OS	[126]
DROSHA	rs6886834 G > A		NSCLC	Reduced RFS	[109]
XPO5	rs11077 A > C		NSCLC	Increased time to recurrence	[108]
			SCLC	Reduced OS	[127]
pri-miR-26a-1	rs7372209 C > T		CRC	Reduced time to progression	[128]
pri-miR-100	rs1834306 T > C		CRC	Increased time to progression	[128]
miR-219-1	rs213210 T > C		CRC	Increased RFS	[129]
			CRC	Increased OS/RFS	[130]
miR-608	rs4919510 C > G		CRC	Decreased RFS	[129]
			CRC	Increased EFS	[130]
KIF3C	rs6728684 T > G		PC	Reduced PFS	[124]
IFI30	rs1045747 T > C		PC	Reduced PFS	[121]
DDX20	rs197412 C > T		BC	Decreased risk of recurrence	[131]
DGCR8	rs2073778 G > T		BC	Increased risk of progression	[131]
XPO5	rs11077 A > C		MM	Increased OS	[119]
			HL	Increased OS/DFS for heterozygotes	[118]
			HCC	Increased OS	[132]
RNASEN	rs17408716 A > G		OC	Better treatment response and increased OS	[116]

^a^ MiRNAs that are not listed as bona fide miRNAs in MirGeneDB [5]. NSCLC: non-small cell lung cancer; SCLC: small cell lung cancer; LC: lung cancer; CRC: colorectal cancer; OC: Ovarian cancer; HCC: hepatocellular carcinoma; HL: Hodgkin lymphoma; MM: multiple myeloma; BC: bladder cancer; PC: prostate cancer.

## References

[1] Baer-Dubowska W., Majchrzak-Celinska A., Cichocki M. (2011). Pharmocoepigenetics: A new approach to predicting individual drug responses and targeting new drugs. Pharmacol. Rep..

[2] Nakajima M., Yokoi T. (2011). MicroRNAs from biology to future pharmacotherapy: Regulation of cytochrome P450s and nuclear receptors. Pharmacol. Ther..

[3] Cascorbi I. (2013). Overlapping effects of genetic variation and epigenetics on drug response: Challenges of pharmacoepigenomics. Pharmacogenomics.

[4] Rukov J.L., Shomron N. (2011). MicroRNA pharmacogenomics: Post-transcriptional regulation of drug response. Trends Mol. Med..

[5] Fromm B., Billipp T., Peck L.E., Johansen M., Tarver J.E., King B.L., Newcomb J.M., Sempere L.F., Flatmark K., Hovig E. (2015). A Uniform System for the Annotation of Vertebrate microRNA Genes and the Evolution of the Human microRNAome. Annu. Rev. Genet..

[6] Friedman R.C., Farh K.K., Burge C.B., Bartel D.P. (2009). Most mammalian mRNAs are conserved targets of microRNAs. Genome Res..

[7] Beermann J., Piccoli M.T., Viereck J., Thum T. (2016). Non-coding RNAs in development and disease: Background, mechanisms, and therapeutic approaches. Physiol. Rev..

[8] He Y., Chevillet J.R., Liu G., Kim T.K., Wang K. (2015). The effects of microRNA on the absorption, distribution, metabolism and excretion of drugs. Br. J. Pharmacol..

[9] Wang L., Oberg A.L., Asmann Y.W., Sicotte H., McDonnell S.K., Riska S.M., Liu W., Steer C.J., Subramanian S., Cunningham J.M. (2009). Genome-wide transcriptional profiling reveals microRNA-correlated genes and biological processes in human lymphoblastoid cell lines. PLoS ONE.

[10] Rieger J.K., Klein K., Winter S., Zanger U.M. (2013). Expression variability of absorption, distribution, metabolism, excretion-related microRNAs in human liver: Influence of nongenetic factors and association with gene expression. Drug Metab. Dispos..

[11] Choi Y.M., An S., Lee E.M., Kim K., Choi S.J., Kim J.S., Jang H.H., An I.S., Bae S. (2012). CYP1A1 is a target of miR-892a-mediated post-transcriptional repression. Int. J. Oncol..

[12] Wang H., Tan W., Hao B., Miao X., Zhou G., He F., Lin D. (2003). Substantial reduction in risk of lung adenocarcinoma associated with genetic polymorphism in CYP2A13, the most active cytochrome P450 for the metabolic activation of tobacco-specific carcinogen NNK. Cancer Res..

[13] Kalscheuer S., Zhang X., Zeng Y., Upadhyaya P. (2008). Differential expression of microRNAs in early-stage neoplastic transformation in the lungs of F344 rats chronically treated with the tobacco carcinogen 4-(methylnitrosamino)-1-(3-pyridyl)-1-butanone. Carcinogenesis.

[14] Tsuchiya Y., Nakajima M., Takagi S., Taniya T., Yokoi T. (2006). MicroRNA regulates the expression of human cytochrome P450 1B1. Cancer Res..

[15] Chang I., Mitsui Y., Fukuhara S., Gill A., Wong D.K., Yamamura S., Shahryari V., Tabatabai Z.L., Dahiya R., Shin D.M. (2015). Loss of miR-200c up-regulates CYP1B1 and confers docetaxel resistance in renal cell carcinoma. Oncotarget.

[16] Devlin A.H., Thompson P., Robson T., McKeown S.R. (2010). Cytochrome P450 1B1 mRNA untranslated regions interact to inhibit protein translation. Mol. Carcinogenes..

[17] Zhang S.Y., Surapureddi S., Coulter S., Ferguson S.S., Goldstein J.A. (2012). Human CYP2C8 is post-transcriptionally regulated by microRNAs 103 and 107 in human liver. Mol. Pharmacol..

[18] Yu D., Green B., Marrone A., Guo Y., Kadlubar S., Lin D., Fuscoe J., Pogribny I., Ning B. (2015). Suppression of CYP2C9 by microRNA hsa-miR-128-3p in human liver cells and association with hepatocellular carcinoma. Sci. Rep..

[19] Yu D., Green B., Tolleson W.H., Jin Y., Mei N., Guo Y., Deng H., Pogribny I., Ning B. (2015). MicroRNA hsa-miR-29a-3p modulates CYP2C19 in human liver cells. Biochem. Pharmacol..

[20] Mohri T., Nakajima M., Fukami T., Takamiya M., Aoki Y., Yokoi T. (2010). Human CYP2E1 is regulated by miR-378. Biochem. Pharmacol..

[21] Shukla U., Tumma N., Gratsch T., Dombkowski A., Novak R.F. (2013). Insights into insulin-mediated regulation of CYP2E1: miR-132/-212 targeting of CYP2E1 and role of phosphatidylinositol 3-kinase, Akt (protein kinase B), mammalian target of rapamycin signaling in regulating miR-132/-212 and miR-122/-181a expression in primary cultured rat hepatocytes. Drug Metab. Dispos..

[22] Chen F., Chen C., Yang S., Gong W., Wang Y., Cianflone K., Tang J., Wang D.W. (2012). Let-7b inhibits human cancer phenotype by targeting cytochrome P450 epoxygenase 2J2. PLoS ONE.

[23] Pan Y.Z., Gao W., Yu A.M. (2009). MicroRNAs regulate CYP3A4 expression via direct and indirect targeting. Drug Metab. Dispos..

[24] Wei Z., Jiang S., Zhang Y., Wang X., Peng X., Meng C., Liu Y., Wang H., Guo L., Qin S. (2014). The effect of microRNAs in the regulation of human CYP3A4: A systematic study using a mathematical model. Sci. Rep..

[25] Vuppalanchi R., Liang T., Goswami C.P., Nalamasu R., Li L., Jones D., Wei R., Liu W., Sarasani V., Janga S.C. (2013). Relationship between differential hepatic microRNA expression and decreased hepatic cytochrome P450 3A activity in cirrhosis. PLoS ONE.

[26] Shi Y., Liu Y., Wei Z., Zhang Y., Zhang L., Jiang S., Xiong Y., Shen L., He L., Xing Q. (2015). Hsa-miR-27a is involved in the regulation of CYP3A4 expression in human livers from Chinese Han population. Pharmacogenomics.

[27] Song K.H., Li T., Owsley E., Chiang J.Y. (2010). A putative role of micro RNA in regulation of cholesterol 7alpha-hydroxylase expression in human hepatocytes. J. Lipid Res..

[28] Chen G., Kim S.H., King A.N., Zhao L., Simpson R.U., Christensen P.J., Wang Z., Thomas D.G., Giordano T.J., Lin L. (2011). CYP24A1 is an independent prognostic marker of survival in patients with lung adenocarcinoma. Clin. Cancer Res..

[29] Komagata S., Nakajima M., Takagi S., Mohri T., Taniya T., Yokoi T. (2009). Human CYP24 catalyzing the inactivation of calcitriol is post-transcriptionally regulated by miR-125b. Mol. Pharmacol..

[30] Mohri T., Nakajima M., Takagi S., Komagata S., Yokoi T. (2009). MicroRNA regulates human vitamin D receptor. Int. J. Cancer.

[31] Choi Y.H., Yu A.M. (2014). ABC transporters in multidrug resistance and pharmacokinetics, and strategies for drug development. Curr. Pharm. Des..

[32] Pan S.T., Li Z.L., He Z.X., Qiu J.X., Zhou S.F. (2016). Molecular mechanisms for tumour resistance to chemotherapy. Clin. Exp. Pharmacol. Physiol..

[33] Boyerinas B., Park S.M., Murmann A.E., Gwin K., Montag A.G., Zillhardt M., Hua Y.J., Lengyel E., Peter M.E. (2012). Let-7 modulates acquired resistance of ovarian cancer to Taxanes via IMP-1-mediated stabilization of multidrug resistance 1. Int. J. Cancer.

[34] Zhu H., Wu H., Liu X., Evans B.R., Medina D.J., Liu C.G., Yang J.M. (2008). Role of MicroRNA miR-27a and miR-451 in the regulation of MDR1/P-glycoprotein expression in human cancer cells. Biochem. Pharmacol..

[35] Li Z., Hu S., Wang J., Cai J., Xiao L., Yu L., Wang Z. (2010). MiR-27a modulates MDR1/P-glycoprotein expression by targeting HIPK2 in human ovarian cancer cells. Gynecol. Oncol..

[36] Bitarte N., Bandres E., Boni V., Zarate R., Rodriguez J., Gonzalez-Huarriz M., Lopez I., Javier Sola J., Alonso M.M., Fortes P. (2011). MicroRNA-451 is involved in the self-renewal, tumorigenicity, and chemoresistance of colorectal cancer stem cells. Stem Cell.

[37] Bao L., Hazari S., Mehra S., Kaushal D., Moroz K., Dash S. (2012). Increased expression of P-glycoprotein and doxorubicin chemoresistance of metastatic breast cancer is regulated by miR-298. Am. J. Pathol..

[38] Yang T., Zheng Z.M., Li X.N., Li Z.F., Wang Y., Geng Y.F., Bai L., Zhang X.B. (2013). MiR-223 modulates multidrug resistance via downregulation of ABCB1 in hepatocellular carcinoma cells. Exp. Biol. Med..

[39] Shang Y., Zhang Z., Liu Z., Feng B., Ren G., Li K., Zhou L., Sun Y., Li M., Zhou J. (2014). MiR-508-5p regulates multidrug resistance of gastric cancer by targeting ABCB1 and ZNRD1. Oncogene.

[40] Ikemura K., Yamamoto M., Miyazaki S., Mizutani H., Iwamoto T., Okuda M. (2013). MicroRNA-145 post-transcriptionally regulates the expression and function of P-glycoprotein in intestinal epithelial cells. Mol. Pharmacol..

[41] Dong Z., Zhong Z., Yang L., Wang S., Gong Z. (2014). MicroRNA-31 inhibits cisplatin-induced apoptosis in non-small cell lung cancer cells by regulating the drug transporter ABCB9. Cancer Lett..

[42] Zhang Y.K., Wang Y.J., Gupta P., Chen Z.S. (2015). Multidrug Resistance Proteins (MRPs) and Cancer Therapy. AAPS J..

[43] Liang Z., Wu H., Xia J., Li Y., Zhang Y., Huang K., Wagar N., Yoon Y., Cho H.T., Scala S. (2010). Involvement of miR-326 in chemotherapy resistance of breast cancer through modulating expression of multidrug resistance-associated protein 1. Biochem. Pharmacol..

[44] Guo L., Liu Y., Bai Y., Sun Y., Xiao F., Guo Y. (2010). Gene expression profiling of drug-resistant small cell lung cancer cells by combining microRNA and cDNA expression analysis. Eur. J. Cancer.

[45] Xu Y., Xia F., Ma L., Shan J., Shen J., Yang Z., Liu J., Cui Y., Bian X., Bie P. (2011). MicroRNA-122 sensitizes HCC cancer cells to adriamycin and vincristine through modulating expression of MDR and inducing cell cycle arrest. Cancer Lett..

[46] Pan Y.Z., Zhou A., Hu Z., Yu A.M. (2013). Small nucleolar RNA-derived microRNA hsa-miR-1291 modulates cellular drug disposition through direct targeting of ABC transporter ABCC1. Drug Metab. Dispos..

[47] Liu H., Wu X., Huang J., Peng J., Guo L. (2015). MiR-7 modulates chemoresistance of small cell lung cancer by repressing MRP1/ABCC1. Int. J. Exp. Pathol..

[48] Haenisch S., Laechelt S., Bruckmueller H., Werk A., Noack A., Bruhn O., Remmler C., Cascorbi I. (2011). Down-regulation of ATP-binding cassette C2 protein expression in HepG2 cells after rifampicin treatment is mediated by microRNA-379. Mol. Pharmacol..

[49] Werk A.N., Bruckmueller H., Haenisch S., Cascorbi I. (2014). Genetic variants may play an important role in mRNA-miRNA interaction: Evidence for haplotype-dependent downregulation of ABCC2 (MRP2) by miRNA-379. Pharmacogenet. Genom..

[50] Xu K., Liang X., Shen K., Cui D., Zheng Y., Xu J., Fan Z., Qiu Y., Li Q., Ni L. (2012). MiR-297 modulates multidrug resistance in human colorectal carcinoma by down-regulating MRP-2. Biochem. J..

[51] Zhan M., Qu Q., Wang G., Zhou H. (2013). Let-7c sensitizes acquired cisplatin-resistant A549 cells by targeting ABCC2 and Bcl-XL. Die Pharm..

[52] Borel F., Han R., Visser A., Petry H., van Deventer S.J., Jansen P.L., Konstantinova P., Réseau Centre de Ressources Biologiques Foie (French Liver Biobanks Network), France (2012). Adenosine triphosphate-binding cassette transporter genes up-regulation in untreated hepatocellular carcinoma is mediated by cellular microRNAs. Hepatology.

[53] Markova S.M., Kroetz D.L. (2014). ABCC4 is regulated by microRNA-124a and microRNA-506. Biochem. Pharmacol..

[54] Zhu Y., Yu F., Jiao Y., Feng J., Tang W., Yao H., Gong C., Chen J., Su F., Zhang Y. (2011). Reduced miR-128 in breast tumor-initiating cells induces chemotherapeutic resistance via Bmi-1 and ABCC5. Clin. Cancer Res..

[55] Robey R.W., Polgar O., Deeken J., To K.W., Bates S.E. (2007). ABCG2: Determining its relevance in clinical drug resistance. Cancer Metast. Rev..

[56] An Y., Ongkeko W.M. (2009). ABCG2: The key to chemoresistance in cancer stem cells?. Expert Opin. Drug Metab. Toxicol..

[57] Pan Y.Z., Morris M.E., Yu A.M. (2009). MicroRNA-328 negatively regulates the expression of breast cancer resistance protein (BCRP/ABCG2) in human cancer cells. Mol. Pharmacol..

[58] Li W.Q., Li Y.M., Tao B.B., Lu Y.C., Hu G.H., Liu H.M., He J., Xu Y., Yu H.Y. (2010). Downregulation of ABCG2 expression in glioblastoma cancer stem cells with miRNA-328 may decrease their chemoresistance. Med. Sci. Monit..

[59] Xu X.T., Xu Q., Tong J.L., Zhu M.M., Nie F., Chen X., Xiao S.D., Ran Z.H. (2012). MicroRNA expression profiling identifies miR-328 regulates cancer stem cell-like SP cells in colorectal cancer. Br. J. Cancer.

[60] Li X., Pan Y.Z., Seigel G.M., Hu Z.H., Huang M., Yu A.M. (2011). Breast cancer resistance protein BCRP/ABCG2 regulatory microRNAs (hsa-miR-328, -519c and -520h) and their differential expression in stem-like ABCG2+ cancer cells. Biochem. Pharmacol..

[61] Turrini E., Haenisch S., Laechelt S., Diewock T., Bruhn O., Cascorbi I. (2012). MicroRNA profiling in K-562 cells under imatinib treatment: Influence of miR-212 and miR-328 on ABCG2 expression. Pharmacogenet. Genom..

[62] Liao R., Sun J., Zhang L., Lou G., Chen M., Zhou D., Chen Z., Zhang S. (2008). MicroRNAs play a role in the development of human hematopoietic stem cells. J. Cell. Biochem..

[63] Wang F., Xue X., Wei J., An Y., Yao J., Cai H., Wu J., Dai C., Qian Z., Xu Z. (2010). Hsa-miR-520h downregulates ABCG2 in pancreatic cancer cells to inhibit migration, invasion, and side populations. Br. J. Cancer.

[64] Jiao X., Zhao L., Ma M., Bai X., He M., Yan Y., Wang Y., Chen Q., Zhao X., Zhou M. (2013). MiR-181a enhances drug sensitivity in mitoxantone-resistant breast cancer cells by targeting breast cancer resistance protein (BCRP/ABCG2). Breast Cancer Res. Treat..

[65] Ma M.T., He M., Wang Y., Jiao X.Y., Zhao L., Bai X.F., Yu Z.J., Wu H.Z., Sun M.L., Song Z.G. (2013). MiR-487a resensitizes mitoxantrone (MX)-resistant breast cancer cells (MCF-7/MX) to MX by targeting breast cancer resistance protein (BCRP/ABCG2). Cancer Lett..

[66] Baudry A., Mouillet-Richard S., Schneider B., Launay J.M., Kellermann O. (2010). MiR-16 targets the serotonin transporter: A new facet for adaptive responses to antidepressants. Science.

[67] Tamarapu Parthasarathy P., Galam L., Huynh B., Yunus A., Abuelenen T., Castillo A., Kollongod Ramanathan G., Cox R., Kolliputi N. (2012). MicroRNA 16 modulates epithelial sodium channel in human alveolar epithelial cells. Biochem. Biophys. Res. Commun..

[68] Moya P.R., Wendland J.R., Salemme J., Fried R.L., Murphy D.L. (2013). MiR-15a and miR-16 regulate serotonin transporter expression in human placental and rat brain raphe cells. Int. J. Neuropsychopharmacol..

[69] Miko E., Margitai Z., Czimmerer Z., Varkonyi I., Dezso B., Lanyi A., Bacso Z., Scholtz B. (2011). MiR-126 inhibits proliferation of small cell lung cancer cells by targeting SLC7A5. FEBS Lett..

[70] Drayton R.M., Dudziec E., Peter S., Bertz S., Hartmann A., Bryant H.E., Catto J.W. (2014). Reduced expression of miRNA-27a modulates cisplatin resistance in bladder cancer by targeting the cystine/glutamate exchanger SLC7A11. Clin. Cancer Res..

[71] Gillen A.E., Gosalia N., Leir S.H., Harris A. (2011). MicroRNA regulation of expression of the cystic fibrosis transmembrane conductance regulator gene. Biochem. J..

[72] Dalmasso G., Nguyen H.T., Yan Y., Laroui H., Charania M.A., Obertone T.S., Sitaraman S.V., Merlin D. (2011). MicroRNA-92b regulates expression of the oligopeptide transporter PepT1 in intestinal epithelial cells. Am. J. Physiol. Gastrointest. Liver Physiol..

[73] Li K.K., Pang J.C., Ching A.K., Wong C.K., Kong X., Wang Y., Zhou L., Chen Z., Ng H.K. (2009). MiR-124 is frequently down-regulated in medulloblastoma and is a negative regulator of SLC16A1. Hum. Pathol..

[74] Pullen T.J., da Silva Xavier G., Kelsey G., Rutter G.A. (2011). MiR-29a and miR-29b contribute to pancreatic beta-cell-specific silencing of monocarboxylate transporter 1 (Mct1). Mol. Cell. Biol..

[75] Kamiyama Y., Matsubara T., Yoshinari K., Nagata K., Kamimura H., Yamazoe Y. (2007). Role of human hepatocyte nuclear factor 4alpha in the expression of drug-metabolizing enzymes and transporters in human hepatocytes assessed by use of small interfering RNA. Drug Metab. Pharm..

[76] Takagi S., Nakajima M., Kida K., Yamaura Y., Fukami T., Yokoi T. (2010). MicroRNAs regulate human hepatocyte nuclear factor 4alpha, modulating the expression of metabolic enzymes and cell cycle. J. Biol. Chem..

[77] Hatziapostolou M., Polytarchou C., Aggelidou E., Drakaki A., Poultsides G.A., Jaeger S.A., Ogata H., Karin M., Struhl K., Hadzopoulou-Cladaras M. (2011). An HNF4alpha-miRNA inflammatory feedback circuit regulates hepatocellular oncogenesis. Cell.

[78] Ramamoorthy A., Li L., Gaedigk A., Bradford L.D., Benson E.A., Flockhart D.A., Skaar T.C. (2012). In silico and in vitro identification of microRNAs that regulate hepatic nuclear factor 4alpha expression. Drug Metab. Dispos..

[79] Wang Z., Burke P.A. (2013). The role of microRNAs in hepatocyte nuclear factor-4alpha expression and transactivation. Biochim. Biophys. Acta.

[80] Takagi S., Nakajima M., Mohri T., Yokoi T. (2008). Post-transcriptional regulation of human pregnane X receptor by micro-RNA affects the expression of cytochrome P450 3A4. J. Biol. Chem..

[81] Wei Z., Chen M., Zhang Y., Wang X., Jiang S., Wang Y., Wu X., Qin S., He L., Zhang L. (2013). No correlation of hsa-miR-148a with expression of PXR or CYP3A4 in human livers from Chinese Han population. PLoS ONE.

[82] Wang Z., Schuetz E.G., Xu Y., Thummel K.E. (2013). Interplay between vitamin D and the drug metabolizing enzyme CYP3A4. J. Steroid Biochem. Mol. Biol..

[83] Keenan C.R., Lew M.J., Stewart A.G. (2016). Biased signalling from the glucocorticoid receptor: Renewed opportunity for tailoring glucocorticoid activity. Biochem. Pharmacol..

[84] Pavek P., Cerveny L., Svecova L., Brysch M., Libra A., Vrzal R., Nachtigal P., Staud F., Ulrichova J., Fendrich Z. (2007). Examination of Glucocorticoid receptor alpha-mediated transcriptional regulation of P-glycoprotein, CYP3A4, and CYP2C9 genes in placental trophoblast cell lines. Placenta.

[85] Vreugdenhil E., Verissimo C.S., Mariman R., Kamphorst J.T., Barbosa J.S., Zweers T., Champagne D.L., Schouten T., Meijer O.C., de Kloet E.R. (2009). MicroRNA 18 and 124a down-regulate the glucocorticoid receptor: Implications for glucocorticoid responsiveness in the brain. Endocrinology.

[86] Adams B.D., Furneaux H., White B.A. (2007). The micro-ribonucleic acid (miRNA) miR-206 targets the human estrogen receptor-alpha (ERalpha) and represses ERalpha messenger RNA and protein expression in breast cancer cell lines. Mol. Endocrinol..

[87] Zhao J.J., Lin J., Yang H., Kong W., He L., Ma X., Coppola D., Cheng J.Q. (2008). MicroRNA-221/222 negatively regulates estrogen receptor alpha and is associated with tamoxifen resistance in breast cancer. J. Biol. Chem..

[88] Pandey D.P., Picard D. (2009). MiR-22 inhibits estrogen signaling by directly targeting the estrogen receptor alpha mRNA. Mol. Cell. Biol..

[89] Xiong J., Yu D., Wei N., Fu H., Cai T., Huang Y., Wu C., Zheng X., Du Q., Lin D. (2010). An estrogen receptor alpha suppressor, microRNA-22, is downregulated in estrogen receptor alpha-positive human breast cancer cell lines and clinical samples. FEBS J..

[90] Tang L., Pu Y., Wong D.K., Liu T., Tang H., Xiang T., Yuen M.F., Ren G. (2011). The hepatitis B virus-associated estrogen receptor alpha (ERalpha) was regulated by microRNA-130a in HepG2.2.15 human hepatocellular carcinoma cells. Acta Biochim. Biophys. Sin..

[91] Zheng L., Lv G.C., Sheng J., Yang Y.D. (2010). Effect of miRNA-10b in regulating cellular steatosis level by targeting PPAR-alpha expression, a novel mechanism for the pathogenesis of NAFLD. J. Gastroenterol. Hepatol..

[92] Kida K., Nakajima M., Mohri T., Oda Y., Takagi S., Fukami T., Yokoi T. (2011). PPARalpha is regulated by miR-21 and miR-27b in human liver. Pharm. Res..

[93] Tong J.L., Zhang C.P., Nie F., Xu X.T., Zhu M.M., Xiao S.D., Ran Z.H. (2011). MicroRNA 506 regulates expression of PPAR alpha in hydroxycamptothecin-resistant human colon cancer cells. FEBS Lett..

[94] Ou Z., Wada T., Gramignoli R., Li S., Strom S.C., Huang M., Xie W. (2011). MicroRNA hsa-miR-613 targets the human LXRalpha gene and mediates a feedback loop of LXRalpha autoregulation. Mol. Endocrinol..

[95] Zhong D., Zhang Y., Zeng Y.J., Gao M., Wu G.Z., Hu C.J., Huang G., He F.T. (2013). MicroRNA-613 represses lipogenesis in HepG2 cells by downregulating LXRalpha. Lipids Health Dis..

[96] Vinod M., Chennamsetty I., Colin S., Belloy L., De Paoli F., Schaider H., Graier W.F., Frank S., Kratky D., Staels B. (2014). MiR-206 controls LXRalpha expression and promotes LXR-mediated cholesterol efflux in macrophages. Biochim. Biophys. Acta.

[97] Yu A.M. (2009). Role of microRNAs in the regulation of drug metabolism and disposition. Expert Opin. Drug Metab. Toxicol..

[98] Rodrigues A.C., Li X., Radecki L., Pan Y.Z., Winter J.C., Huang M., Yu A.M. (2011). MicroRNA expression is differentially altered by xenobiotic drugs in different human cell lines. Biopharm. Drug Dispos..

[99] Pogribny I.P., Filkowski J.N., Tryndyak V.P., Golubov A., Shpyleva S.I., Kovalchuk O. (2010). Alterations of microRNAs and their targets are associated with acquired resistance of MCF-7 breast cancer cells to cisplatin. Int. J. Cancer.

[100] Takahashi K., Tatsumi N., Fukami T., Yokoi T., Nakajima M. (2014). Integrated analysis of rifampicin-induced microRNA and gene expression changes in human hepatocytes. Drug Metab. Pharm..

[101] Benson E.A., Eadon M.T., Desta Z., Liu Y., Lin H., Burgess K.S., Segar M.W., Gaedigk A., Skaar T.C. (2016). Rifampin Regulation of Drug Transporters Gene Expression and the Association of MicroRNAs in Human Hepatocytes. Front. Pharmacol..

[102] Liu C., Zhang F., Li T., Lu M., Wang L., Yue W., Zhang D. (2012). MirSNP, a database of polymorphisms altering miRNA target sites, identifies miRNA-related SNPs in GWAS SNPs and eQTLs. BMC Genom..

[103] Dzikiewicz-Krawczyk A. (2015). MicroRNA polymorphisms as markers of risk, prognosis and treatment response in hematological malignancies. Crit. Rev. Oncol. Hematol..

[104] Siegel R., Naishadham D., Jemal A. (2012). Cancer statistics, 2012. CA Cancer J. Clin..

[105] Ding C., Li R., Peng J., Li S., Guo Z. (2012). A polymorphism at the miR-502 binding site in the 3’ untranslated region of the SET8 gene is associated with the outcome of small-cell lung cancer. Exp. Ther. Med..

[106] Xu J., Yin Z., Gao W., Liu L., Yin Y., Liu P., Shu Y. (2013). Genetic variation in a microRNA-502 minding site in SET8 gene confers clinical outcome of non-small cell lung cancer in a Chinese population. PLoS ONE.

[107] Cheng M., Yang L., Yang R., Yang X., Deng J., Yu B., Huang D., Zhang S., Wang H., Qiu F. (2013). A microRNA-135a/b binding polymorphism in CD133 confers decreased risk and favorable prognosis of lung cancer in Chinese by reducing CD133 expression. Carcinogenesis.

[108] Campayo M., Navarro A., Vinolas N., Tejero R., Munoz C., Diaz T., Marrades R., Cabanas M.L., Gimferrer J.M., Gascon P. (2011). A dual role for KRT81: A miR-SNP associated with recurrence in non-small-cell lung cancer and a novel marker of squamous cell lung carcinoma. PLoS ONE.

[109] Pu X., Roth J.A., Hildebrandt M.A., Ye Y., Wei H., Minna J.D., Lippman S.M., Wu X. (2013). MicroRNA-related genetic variants associated with clinical outcomes in early-stage non-small cell lung cancer patients. Cancer Res..

[110] Lin J., Zandi R., Gu J., Ye Y.Q., Pertsemlidis A., Wu X.F., Roth J.A., Ji L. (2015). A SNP in the 3’-untranslated region of FZD4 linked to lung cancer survival modulates a miRNA-mediated FZD4 transcript binding, cleavage, expression, and Wnt-signaling in NSCLC cells. Cancer Res..

[111] Graziano F., Canestrari E., Loupakis F., Ruzzo A., Galluccio N., Santini D., Rocchi M., Vincenzi B., Salvatore L., Cremolini C. (2010). Genetic modulation of the Let-7 microRNA binding to KRAS 3’-untranslated region and survival of metastatic colorectal cancer patients treated with salvage cetuximab-irinotecan. Pharmacogenom. J..

[112] Sebio A., Pare L., Paez D., Salazar J., Gonzalez A., Sala N., del Rio E., Martin-Richard M., Tobena M., Barnadas A. (2013). The LCS6 polymorphism in the binding site of let-7 microRNA to the KRAS 3’-untranslated region: Its role in the efficacy of anti-EGFR-based therapy in metastatic colorectal cancer patients. Pharmacogenet. Genom..

[113] Zhang W., Winder T., Ning Y., Pohl A., Yang D., Kahn M., Lurje G., Labonte M.J., Wilson P.M., Gordon M.A. (2011). A let-7 microRNA-binding site polymorphism in 3’-untranslated region of KRAS gene predicts response in wild-type KRAS patients with metastatic colorectal cancer treated with cetuximab monotherapy. Ann. Oncol..

[114] Kjersem J.B., Ikdahl T., Guren T., Skovlund E., Sorbye H., Hamfjord J., Pfeiffer P., Glimelius B., Kersten C., Solvang H. (2012). Let-7 miRNA-binding site polymorphism in the KRAS 3'UTR; colorectal cancer screening population prevalence and influence on clinical outcome in patients with metastatic colorectal cancer treated with 5-fluorouracil and oxaliplatin +/− cetuximab. BMC Cancer.

[115] Wynendaele J., Bohnke A., Leucci E., Nielsen S.J., Lambertz I., Hammer S., Sbrzesny N., Kubitza D., Wolf A., Gradhand E. (2010). An illegitimate microRNA target site within the 3’ UTR of MDM4 affects ovarian cancer progression and chemosensitivity. Cancer Res..

[116] Liang D., Meyer L., Chang D.W., Lin J., Pu X., Ye Y., Gu J., Wu X., Lu K. (2010). Genetic variants in MicroRNA biosynthesis pathways and binding sites modify ovarian cancer risk, survival, and treatment response. Cancer Res..

[117] Guo Z., Wu C., Wang X., Wang C., Zhang R., Shan B. (2012). A polymorphism at the miR-502 binding site in the 3’-untranslated region of the histone methyltransferase SET8 is associated with hepatocellular carcinoma outcome. Int. J. Cancer.

[118] Navarro A., Munoz C., Gaya A., Diaz-Beya M., Gel B., Tejero R., Diaz T., Martinez A., Monzo M. (2013). MiR-SNPs as markers of toxicity and clinical outcome in Hodgkin lymphoma patients. PLoS ONE.

[119] De Larrea C.F., Navarro A., Tejero R., Tovar N., Diaz T., Cibeira M.T., Rosinol L., Ferrer G., Rovira M., Rozman M. (2012). Impact of MiRSNPs on survival and progression in patients with multiple myeloma undergoing autologous stem cell transplantation. Clin. Cancer Res..

[120] Wu Y., Xiao Y., Ding X., Zhuo Y., Ren P., Zhou C., Zhou J. (2011). A miR-200b/200c/429-binding site polymorphism in the 3’ untranslated region of the AP-2alpha gene is associated with cisplatin resistance. PLoS ONE.

[121] Bao B.Y., Pao J.B., Huang C.N., Pu Y.S., Chang T.Y., Lan Y.H., Lu T.L., Lee H.Z., Juang S.H., Chen L.M. (2011). Polymorphisms inside microRNAs and microRNA target sites predict clinical outcomes in prostate cancer patients receiving androgen-deprivation therapy. Clin. Cancer Res..

[122] Huang S.P., Levesque E., Guillemette C., Yu C.C., Huang C.Y., Lin V.C., Chung I.C., Chen L.C., Laverdiere I., Lacombe L. (2014). Genetic variants in microRNAs and microRNA target sites predict biochemical recurrence after radical prostatectomy in localized prostate cancer. Int. J. Cancer.

[123] Zhan X., Wu W., Han B., Gao G., Qiao R., Lv J., Zhang S., Zhang W., Fan W., Chen H. (2012). Hsa-miR-196a2 functional SNP is associated with severe toxicity after platinum-based chemotherapy of advanced nonsmall cell lung cancer patients in a Chinese population. J. Clin. Lab. Anal..

[124] Hong M.J., Choi Y.Y., Jang J.A., Jung H.J., Lee S.Y., Lee W.K., Yoo S.S., Lee J., Cha S.I., Kim C.H. (2013). Association between genetic variants in pre-microRNAs and survival of early-stage NSCLC. J. Thorac. Oncol..

[125] Xu J., Yin Z., Shen H., Gao W., Qian Y., Pei D., Liu L., Shu Y. (2013). A genetic polymorphism in pre-miR-27a confers clinical outcome of non-small cell lung cancer in a Chinese population. PLoS ONE.

[126] Zhao Y., Wei Q., Hu L., Chen F., Hu Z., Heist R.S., Su L., Amos C.I., Shen H., Christiani D.C. (2014). Polymorphisms in MicroRNAs are associated with survival in non-small cell lung cancer. Cancer Epidemiol. Biomarkers Prev..

[127] Guo Z., Wang H., Li Y., Li B., Li C., Ding C. (2013). A microRNA-related single nucleotide polymorphism of the XPO5 gene is associated with survival of small cell lung cancer patients. Biomed. Rep..

[128] Boni V., Zarate R., Villa J.C., Bandres E., Gomez M.A., Maiello E., Garcia-Foncillas J., Aranda E. (2011). Role of primary miRNA polymorphic variants in metastatic colon cancer patients treated with 5-fluorouracil and irinotecan. Pharmacogenom. J..

[129] Lin M., Gu J., Eng C., Ellis L.M., Hildebrandt M.A., Lin J., Huang M., Calin G.A., Wang D., Dubois R.N. (2012). Genetic polymorphisms in MicroRNA-related genes as predictors of clinical outcomes in colorectal adenocarcinoma patients. Clin. Cancer Res..

[130] Pardini B., Rosa F., Naccarati A., Vymetalkova V., Ye Y., Wu X., di Gaetano C., Buchler T., Novotny J., Matullo G. (2015). Polymorphisms in microRNA genes as predictors of clinical outcomes in colorectal cancer patients. Carcinogenesis.

[131] Ke H.L., Chen M., Ye Y., Hildebrandt M.A., Wu W.J., Wei H., Huang M., Chang D.W., Dinney C.P., Wu X. (2013). Genetic variations in micro-RNA biogenesis genes and clinical outcomes in non-muscle-invasive bladder cancer. Carcinogenesis.

[132] Liu S., An J., Lin J., Liu Y., Bao L., Zhang W., Zhao J.J. (2014). Single nucleotide polymorphisms of microRNA processing machinery genes and outcome of hepatocellular carcinoma. PLoS ONE.

[133] Henrichsen C.N., Chaignat E., Reymond A. (2009). Copy number variants, diseases and gene expression. Hum. Mol. Genet..

[134] Duan S., Mi S., Zhang W., Dolan M.E. (2009). Comprehensive analysis of the impact of SNPs and CNVs on human microRNAs and their regulatory genes. RNA Biol..

[135] Lian J., Zhang X., Tian H., Liang N., Wang Y., Liang C., Li X., Sun F. (2009). Altered microRNA expression in patients with non-obstructive azoospermia. Reprod. Biol. Endocrinol..

[136] Keller A., Leidinger P., Lange J., Borries A., Schroers H., Scheffler M., Lenhof H.P., Ruprecht K., Meese E. (2009). Multiple sclerosis: microRNA expression profiles accurately differentiate patients with relapsing-remitting disease from healthy controls. PLoS ONE.

[137] Marrale M., Albanese N.N., Cali F., Romano V. (2014). Assessing the impact of copy number variants on miRNA genes in autism by Monte Carlo simulation. PLoS ONE.

[138] Marcinkowska M., Szymanski M., Krzyzosiak W.J., Kozlowski P. (2011). Copy number variation of microRNA genes in the human genome. BMC Genom..

[139] Han M., Zheng Y. (2013). Comprehensive analysis of single nucleotide polymorphisms in human microRNAs. PLoS ONE.

[140] Rawlings-Goss R.A., Campbell M.C., Tishkoff S.A. (2014). Global population-specific variation in miRNA associated with cancer risk and clinical biomarkers. BMC Med. Genom..

[141] Lu J., Clark A.G. (2012). Impact of microRNA regulation on variation in human gene expression. Genome Res..

[142] Huang R.S., Gamazon E.R., Ziliak D., Wen Y., Im H.K., Zhang W., Wing C., Duan S., Bleibel W.K., Cox N.J. (2011). Population differences in microRNA expression and biological implications. RNA Boil..

[143] Bovell L.C., Shanmugam C., Putcha B.D., Katkoori V.R., Zhang B., Bae S., Singh K.P., Grizzle W.E., Manne U. (2013). The prognostic value of microRNAs varies with patient race/ethnicity and stage of colorectal cancer. Clin. Cancer Res..

[144] Chang X., Li S., Li J., Yin L., Zhou T., Zhang C., Chen X., Sun K. (2014). Ethnic differences in microRNA-375 expression level and DNA methylation status in type 2 diabetes of Han and Kazak populations. J. Diabetes Res..

[145] Wang X., Sundquist J., Zoller B., Memon A.A., Palmer K., Sundquist K., Bennet L. (2014). Determination of 14 circulating microRNAs in Swedes and Iraqis with and without diabetes mellitus type 2. PLoS ONE.

[146] Li E., Ji P., Ouyang N., Zhang Y., Wang X.Y., Rubin D.C., Davidson N.O., Bergamaschi R., Shroyer K.R., Burke S. (2014). Differential expression of miRNAs in colon cancer between African and Caucasian Americans: Implications for cancer racial health disparities. Int. J. Oncol..

